# FAIR High Content Screening in Bioimaging

**DOI:** 10.1038/s41597-023-02367-w

**Published:** 2023-07-17

**Authors:** Rohola Hosseini, Matthijs Vlasveld, Joost Willemse, Bob van de Water, Sylvia E. Le Dévédec, Katherine J. Wolstencroft

**Affiliations:** 1 Life Science Semantics, Leiden Institute of Advanced Computer Science, Leiden, The Netherlands; 2 Drug Discovery and Safety, Cell Observatory, Leiden Academic Centre for Drug Research, Leiden, The Netherlands; 3 Cell Observatory, Institute of Biology Leiden, Leiden, The Netherlands

**Keywords:** Molecular imaging, Research data

## Abstract

The Minimum Information for High Content Screening Microscopy Experiments (MIHCSME) is a metadata model and reusable tabular template for sharing and integrating high content imaging data. It has been developed by combining the ISA (Investigations, Studies, Assays) metadata standard with a semantically enriched instantiation of REMBI (Recommended Metadata for Biological Images). The tabular template provides an easy-to-use practical implementation of REMBI, specifically for High Content Screening (HCS) data. In addition, ISA compliance enables broader integration with other types of experimental data, paving the way for visual omics and multi-Omics integration. We show the utility of MIHCSME for HCS data using multiple examples from the Leiden FAIR Cell Observatory, a Euro-Bioimaging flagship node for high content screening and the pilot node for implementing Findable, Accessible, Interoperable and Reusable (FAIR) bioimaging data throughout the Netherlands Bioimaging network.

## Introduction

The recently published recommendations for describing bio-imaging experiments, REMBI (Recommended Metadata for Biological Images)^1^ enable the bioimaging community to improve the archiving and reuse of bioimaging data. The recommendations are supported by public repositories, such as the Image Data Resource (IDR)^2^, which is based on the OMERO database^3^, and were created as a community initiative to encompass a broad range of bioimaging use cases.

The introduction of REMBI is timely for bioimaging, and for a broader biological audience. The importance of FAIR data (i.e., data that is Findable, Accessible, Interoperable and Reusable)^4^ is widely recognised across the life sciences^5^. Microscopy research has lagged-behind other disciplines in data management and sharing, which hampers efforts for integration. However, recent advances in spatio-temporal omics techniques, and the further integration of multiomics readouts of high-throughput experiments, motivate a closer integration of bioimaging and multiomics data. As REMBI focuses exclusively on bioimaging, the context of HCS results in relation to associated omics or modelling results may be lost. The established ISA metadata framework can be used to address this problem^6^. By combining REMBI metadata with ISA, we can enable FAIRer sharing of bioimaging data. Orthogonal developments from the Open Microscopy Environment consortium^7^ provide image format interoperability and exchange, with dedicated image data storage in OMERO.

REMBI is an example of a *minimum information model*, following a similar paradigm to others, such as, the *Minimum Information About a Microarray Experiment (MIAME)*, which was the first to be proposed^8^. Successful uptake of these models shows that the recommendations are an important part of the standardisation process and enable engagement with the user community. However, for widespread uptake, additional tooling is required to provide the recommendations in easy-to-use formats that can be adopted by the community, as demonstrated by the uptake of the tabular format of MIAME, MAGE-TAB^9^. In many cases, this requires general recommendations to be further constrained to serve the purposes of specific types of data. The evolution of MIAME to MINSEQE (Minimum Information about a high-throughput Nucleotide SeQuencing Experiment)^10^, to better describe transcriptomics from RNA-Seq demonstrates this point.

For REMBI, the task of defining common metadata recommendations is arguably much larger than in the case of transcriptomics. The REMBI consortium was divided into three working groups as a reflection of the heterogeneity of biological scales and biological imaging methods. Common metadata requirements were identified across all working groups, but at the expense of detailed specifications for any individual working group. REMBI provides a necessary common point of reference, developed for the community, by the community. It does not offer a reusable metadata format, but a set of guidelines from which metadata templates may be developed. To use the REMBI recommendations, users must first interpret the REMBI guidelines, for their use-case, and then instantiate them. Here we demonstrate the *added value* of creating more specific and targeted instantiations of REMBI, with integrated ISA metadata elements, for practical adoption by data producers. We use high content screening as an example bioimaging research domain (represented by REMBI working group 3) and we provide the resulting MIHCSME format as a reusable tabular template.

The templates serve multiple purposes:

- An example of information that should be collected for a high content screening experiment
- An instantiation of a REMBI-compliant format
- A mechanism for easier import to public repositories, such as the IDR
- Integration with the existing ISA framework (Investigations, Studies and Assays), to enable aggregation of experiment descriptions within bioimaging and with omics analyses
- A mechanism for easier exchange and comparison between similar data sets and to enable export to other formats, such as ISATab or RDF/Linked Data

## Leiden FAIR Bioimaging Data Sharing

The Leiden FAIR Cell Observatory (LFCO) is a Flagship node for high content screening in Euro-bioimaging. It also leads the data management and analysis activities of the Dutch national bioimaging network, NL-Bioimaging^11^. As such, the LFCO has developed a FAIR (Findable, Accessible, Interoperable and Reusable) data management framework for sharing and reusing data, which builds on the OMERO database and the REMBI and ISA metadata standards. LFCO members have been involved in the development of REMBI guidelines.

OMERO is specifically designed to manage data from bioimaging experiments and has become the ‘de facto’ standard for storing bioimaging data, with numerous installations globally. The Image Data Resource (https://idr.openmicroscopy.org/), for example, is a public instance of OMERO for sharing high-quality, published datasets with the community.

The Open Microscopy Environment standard (OME), which OMERO is based on, enables images from different microscope manufacturers to be ingested and exchanged in common formats. Image acquisition metadata is automatically collected and stored on upload and additional plugins enable links between data acquisition and image analysis. Metadata describing the bioimaging experiments, however, and the image analysis processes enacted on the data, are limited to key value pairs in the current version of OMERO. Methods for collecting and sharing standards-compliant metadata are being further developed in NL-Bioimaging, for sharing more broadly with the Euro-Bioimaging and Global-Bioimaging communities. The results of the pilot implementation at the LFCO show that combining OME standards with REMBI and ISA-compliant experimental metadata results in an OMERO database that fulfils the FAIR principles. This enables greater reuse of data in the bioimaging community and greater interoperability with other FAIR life science resources, such as those recommended by ELIXIR^12^.

## Specifying Minimal Metadata Requirements for High Content Screening

**Fig. 1:**
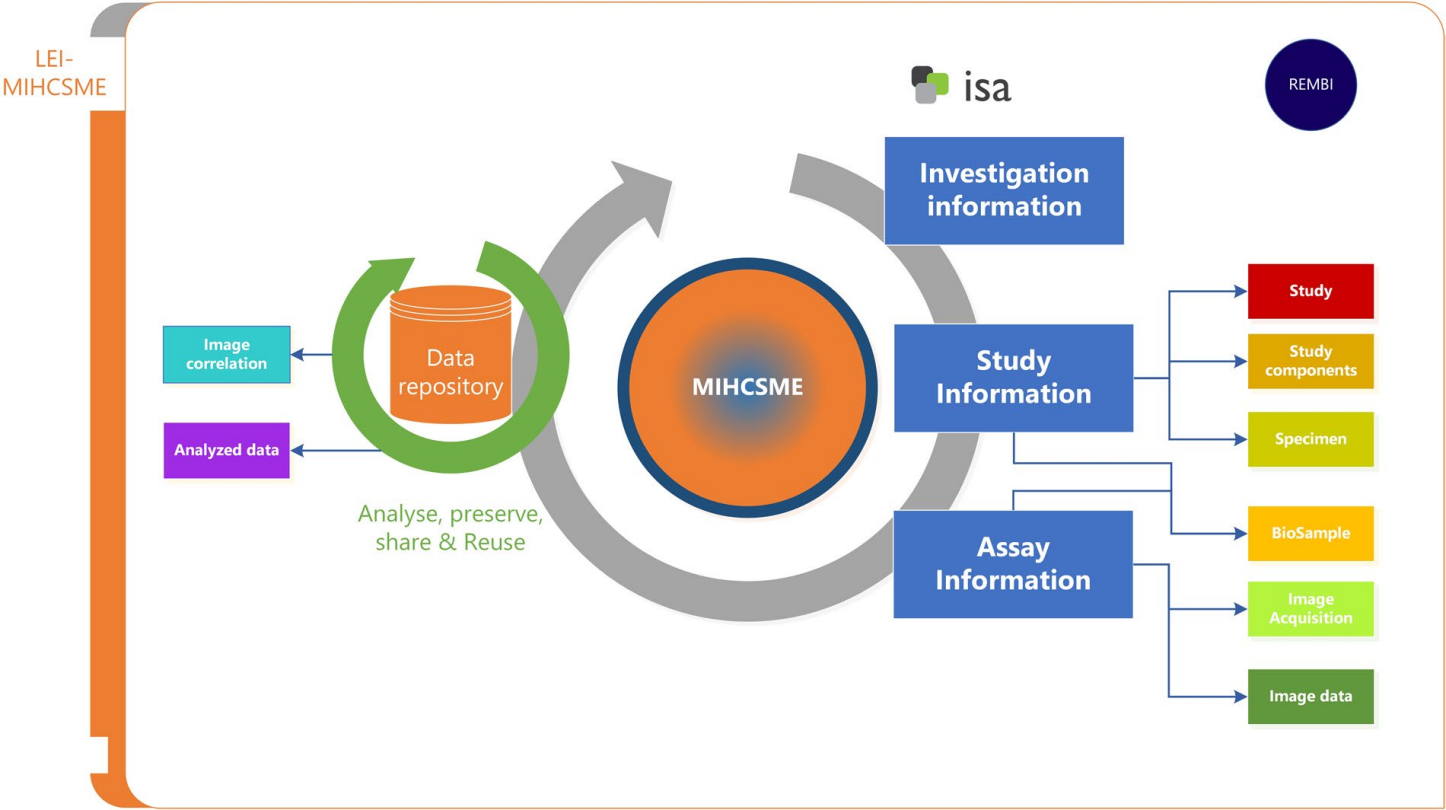
Investigation Design Map for a MIHCSME template and correspondence to the REMBI and ISA recommendations.

REMBI and ISA both encompass the bioimaging data life cycle, from the study design, laboratory preparation of samples and specimens, to data acquisition, processing and analysis. Figure 1 shows an Investigation Design Map (IDM) of how ISA and REMBI metadata are integrated into MIHCSME. The IDM highlights the complex nature of a high content screening experiment and the interdependencies between different life cycle components. We have increased the semantic richness of the metadata by specifying not only which ontologies to adopt as annotation vocabularies, but also the ranges of terms that should be used for annotation of specific fields.

In the REMBI publication, Working Group 3 present an example data set for high content screening. We present the same dataset in the MIHCSME format, showing the additional constraints that lead to a richer description of experiments, including ranges of choices required from recommended ontologies and controlled vocabularies for specific fields (Table 1 and https://fairdomhub.org/assays/2041).

**Table 1:** Examples of HCS data provided with MIHCSME metadata.

Investigation	Availability	Template Link
Compound screen on HepG2 CHOP-GFP reporter	Pre-publication at LFCO	https://fairdomhub.org/assays/2053
Integration of biological data by kernels on graph nodes allows prediction of new genes involved in mitotic chromosome condensation.	Published in IDR https://idr.openmicroscopy.org/webclient/?show=screen-102	https://fairdomhub.org/assays/2041
Uncovering the signaling landscape controlling breast cancer cell migration identifies novel metastasis driver genes	Published in IDR https://idr.openmicroscopy.org/webclient/?show=screen-2151	https://fairdomhub.org/assays/2040
Live cell imaging 72-hour screening	Published in BioStudies https://www.ebi.ac.uk/biostudies/eu-toxrisk/studies/S-TOXR1741	https://fairdomhub.org/assays/2027

REMBI recommends the use of controlled vocabularies, such as, EDAM-Bioimaging^13^ and the Experimental Factor Ontology^14^. ISA takes a similar approach. For some fields, users are asked to select from multiple recommended vocabularies. Whilst this makes the metadata schema flexible for widespread use, it presents practical difficulties for bioimaging specialists unfamiliar with these vocabularies. By constraining the choice for a particular community, we make the process of representing data in a standard way more accessible. This should increase the ease of use, reduce errors and therefore encourage uptake.

This work builds on previous approaches developed during the FAIRDOM project^15^, where providing tabular metadata templates that could be completed in Excel or Open office were found to provide the lowest barrier for entry to semantic data collection. Furthermore, it enabled the automated extraction and transformation into Linked Data, for improved interoperability with related resources. The JERM (Just Enough Results Model) Ontology was used as an application ontology to facilitate links between minimum information models and ISA^16^. We follow the same approach with MIHCSME.

MIHCSME has been tested and formally adopted by high content screening researchers in NL-Bioimaging and the Euro-bioimaging HCS Flagship node. The MIHCSME metadata template is available for download from the FAIRDOMHub (https://fairdomhub.org/investigations/575). The MIHCSME specification is more constrained than the metadata requirements for both the IDR and the BioStudies^17^ (a general repository for sharing biological study information), streamlining the process of public repository submission and archiving.

Here, we present four different examples of MIHCSME in use, showing its utility across HCS (Table 1). The MIHCSME template, and examples with data values, are available for download from the FAIRDOMHub (https://fairdomhub.org/investigations/575). MIHCSME metadata contains additional information than that required by IDR and BioStudies. These public repositories are designed for data reuse but omit some of the data reproducibility elements that are included in ISA. Combining the two approaches provides the same metadata with additional richness to improve reproducibility and interoperability. For example, MIHCSME provides more details on each imaging channel and what is visualised per channel. In addition, screens are represented as assays, which are linked to a broader study that should also include other assays detailing the set-up of the screen (e.g. concentration series), or validation experiments (e.g. qPCR). In REMBI (and the IDR) a screen is itself a study and is presented without this supporting information. More importantly, MIHCSME assists with interlinking data. For example, the Biostudies HCS dataset was used as the basis of a mathematical model which has been published in the BioModels repository^18^ (https://www.ebi.ac.uk/biomodels/MODEL2206070001). With the integration of ISA in MIHCSME, it is possible to describe and maintain this link. This shows the importance of standardisation for the interoperability of data across multiple life science domains. Further details of the commonalities and differences between MIHCSME, REMBI and IDR metadata can be found at (https://fairdomhub.org/sops/587).

**Fig. 2:**
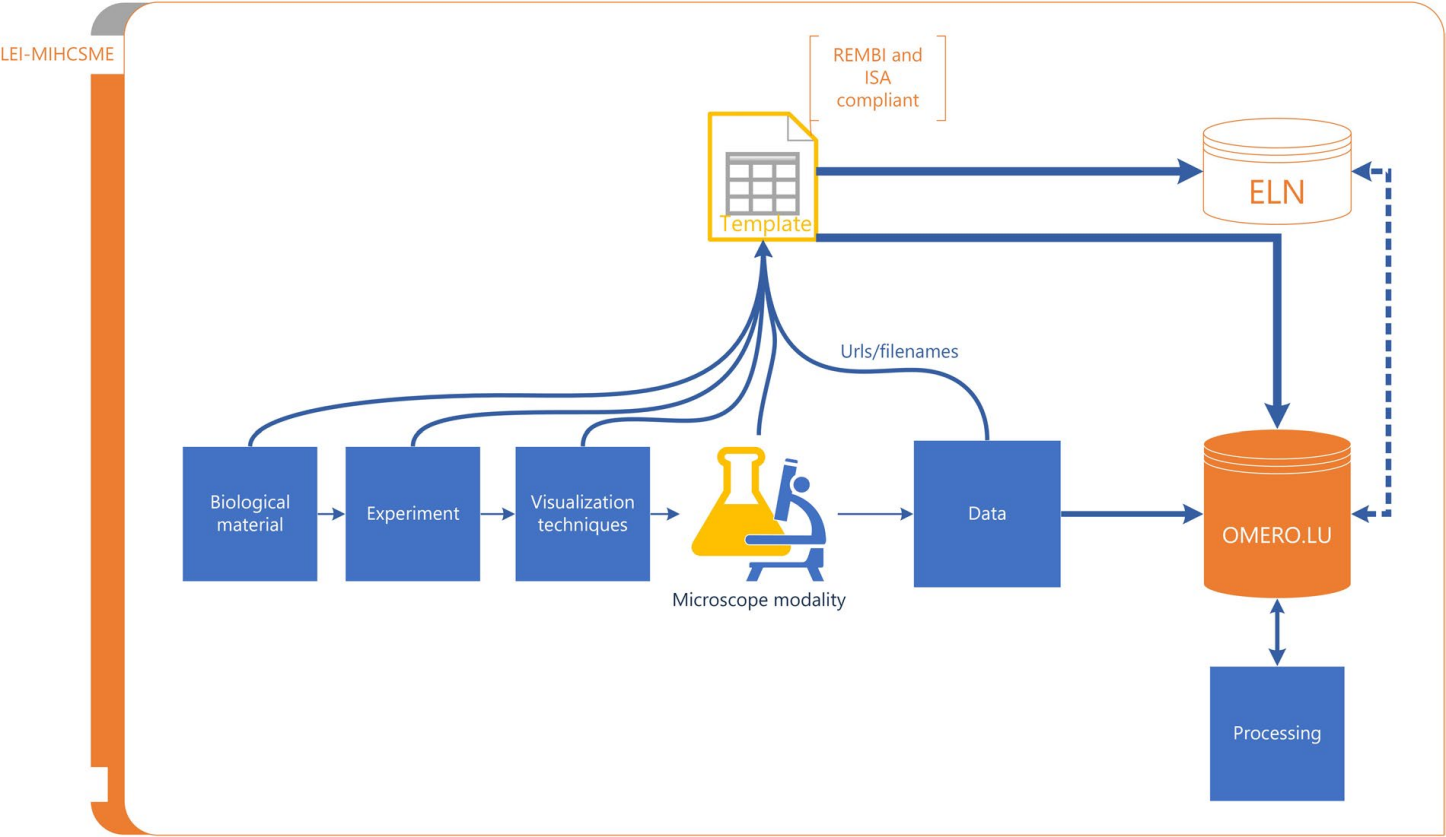
Schematic workflow of HCS microscopy data storage, and metadata recorded in a MIHCSME template.

At the LFCO, researchers upload data to the OMERO database, metadata automatically generated by the microscopes, and MIHCSME metadata created to describe their experiments. OMERO has been interlinked with the local Electronic Lab Notebook to ensure data and metadata are stored and shared in context. In the future, uploading MIHCSME-compliant metadata to OMERO will be required before microscopes can be used for image acquisition. This way of working is the best-practice recommendation for the whole NL-Bioimaging network. Figure 2 shows the user workflow for data and metadata at the LFCO.

## Discussion

The adoption of metadata standards is dependent upon a clear description of what is required, and a method of implementation that does not add a large overhead for individual researchers. The introduction of REMBI was a catalyst for improving the reporting of bioimaging metadata through community consensus, but still requires individual organisations or researchers to understand and implement the recommendations in a manner suitable for their bioimaging domain. The introduction of REMBI-Compliant MIHCSME templates is a practical way of reducing the barrier to adoption. The additional integration of the ISA specification, for organising and interlinking multiple types of data broadens the applicability of REMBI and facilitates collaboration. Templates can be directly used by researchers as they gather their data, increasing the interoperability and reusability of the data without requiring extra data management time or expertise.

Progress towards FAIR bioimaging data (or FAIR data more generally) can be achieved by targeting points in the data management life cycle that require more attention. Most approaches focus on *FAIRification for publication*, which typically occurs at the end of projects. In NL-Bioimaging, we focus on improving the FAIRness of projects from data acquisition onwards. By enabling the scientists who create data to standardise it and improve its FAIRness from the start, we reduce reporting errors, enable and encourage greater collaboration within projects, and make FAIR data sharing easier during publication. The next steps for NL-Bioimaging are to produce similar templates for other *specific* bioimaging domains and to share them with the global bioimaging community. Increasing the adoption of such templates, promotes standardisation and the increased usage can help to drive the future development of REMBI, ISA and related standards.
